# Comparative Cost of Early Infant Male Circumcision by Nurse-Midwives and Doctors in Zimbabwe

**DOI:** 10.9745/GHSP-D-15-00201

**Published:** 2016-07-02

**Authors:** Collin Mangenah, Webster Mavhu, Karin Hatzold, Andrea K Biddle, Getrude Ncube, Owen Mugurungi, Ismail Ticklay, Frances M Cowan, Harsha Thirumurthy

**Affiliations:** a Centre for Sexual Health and HIV/AIDS Research (CeSHHAR), Harare, Zimbabwe; b University College London, London, United Kingdom; c Population Services International, Harare, Zimbabwe; d University of North Carolina at Chapel Hill, Chapel Hill, NC, USA; e Ministry of Health and Child Care, Harare, Zimbabwe; f University of Zimbabwe College of Health Sciences, Harare, Zimbabwe

## Abstract

**Background:**

The 14 countries that are scaling up voluntary male medical circumcision (VMMC) for HIV prevention are also considering early infant male circumcision (EIMC) to ensure longer-term reductions in HIV incidence. The cost of implementing EIMC is an important factor in scale-up decisions. We conducted a comparative cost analysis of EIMC performed by nurse-midwives and doctors using the AccuCirc device in Zimbabwe.

**Methods:**

Between August 2013 and July 2014, nurse-midwives performed EIMC on 500 male infants using AccuCirc in a field trial. We analyzed the overall unit cost and identified key cost drivers of EIMC performed by nurse-midwives and compared these with costing data previously collected during a randomized noninferiority comparison trial of 2 devices (AccuCirc and the Mogen clamp) in which doctors performed EIMC. We assessed direct costs (consumable and nonconsumable supplies, device, personnel, associated staff training, and waste management costs) and indirect costs (capital and support personnel costs). We performed one-way sensitivity analyses to assess cost changes when we varied key component costs.

**Results:**

The unit costs of EIMC performed by nurse-midwives and doctors in vertical programs were US$38.87 and US$49.77, respectively. Key cost drivers of EIMC were consumable supplies, personnel costs, and the device price. In this cost analysis, major cost drivers that explained the differences between EIMC performed by nurse-midwives and doctors were personnel and training costs, both of which were lower for nurse-midwives.

**Conclusions:**

EIMC unit costs were lower when performed by nurse-midwives compared with doctors. To minimize costs, countries planning to scale up EIMC should consider using nurse-midwives, who are in greater supply than doctors and are the main providers at the primary health care level, where most infants are born.

## Abstract (precis)

Early infant male circumcision (EIMC) conducted by nurse-midwives using the AccuCirc device was safe and less costly per procedure than when conducted by doctors: for nurse-midwives, US$38.87 in vertical programs and US$33.72 in integrated programs; for doctors, US$49.77 in vertical programs.

## INTRODUCTION

Based on evidence that medical male circumcision reduces the risk of HIV acquisition among men by up to 60% and is a cost-effective intervention,^1^^-^^8^ a number of sub-Saharan African countries are considering offering early infant male circumcision (EIMC) services in parallel with existing voluntary medical male circumcision (VMMC) services that are typically for 15- to 49-year-old adolescents and adults. While the HIV prevention benefits from EIMC would not be realized immediately, increased uptake of EIMC could reduce the need for adult male circumcision in future years. The World Health Organization (WHO) and the United Nations Children’s Fund (UNICEF) recommend EIMC—performed within the first 60 days of life—for HIV prevention in countries with high HIV prevalence.^9^^,^^10^ EIMC has several advantages over VMMC, including being a more easily performed procedure with faster wound healing and lower rates of adverse events.^11^^,^^12^ In Zimbabwe and other sub-Saharan African countries that are most affected by HIV/AIDS, however, EIMC scale-up may be hindered by an acute shortage of human resources for health, particularly doctors. The shortage of doctors has been reported elsewhere as being one of the most important supply-side barriers to scaling up VMMC.^13^

In Zimbabwe and other sub-Saharan African countries, EIMC scale-up may be hindered by an acute shortage of human resources for health, particularly medical doctors.

Nurse-midwives are nurses trained to manage women's health care, particularly pregnancy, childbirth, the postpartum period, care of the newborn, and gynecology. For countries planning to roll out EIMC, using nurse-midwives to perform the procedure instead of doctors may be a solution. Nurse-midwives are able to provide comparably high-quality, affordable primary care.^14^ Studies have shown that with adequate training, nurse-for-doctor substitution is feasible for many health care interventions. Patient outcomes for trained nurse-midwives are similar to those for doctors.^15^ In a meta-analysis of VMMC performed by trained medical staff such as nurse-midwives, surgical aides, and clinical officers, the frequency of adverse events was similar to that of doctors or specialists.^13^ Substituting nurse-midwives for doctors not only provides solutions for the scarcity of doctors in resource-limited settings, but may also help to contain costs because personnel costs are lower.^16^^,^^17^

In relation to substituting nurse-midwives for doctors for medical male circumcision in general and EIMC in particular, there is a dearth of information on quality, outcomes, and costs. This situation is exacerbated by the fact that in some sub-Saharan African countries, including Zimbabwe, ministry of health guidelines preclude nurse-midwives from performing EIMC with devices that are currently in use, such as the Gomco clamp, Plastibell, and the Mogen clamp.^11^ A relatively new EIMC device, AccuCirc (introduced in 2008), has potential advantages for resource-limited settings^18^ since it requires less surgical skill than the other devices and can thus be more easily used by nurse-midwives.

AccuCirc (introduced in 2008) requires less surgical skill than the Mogen clamp and can thus be more easily used by nurse–midwives.

In this study, we report results from a cost analysis of EIMC performed by nurse-midwives and compare them with costs estimated in a previous cost analysis of EIMC performed by doctors using the AccuCirc and the Mogen clamp in the same study setting in Zimbabwe.^19^ Because opportunities for cost savings may also exist in integrated primary care settings where staff, training, and other costs are shared across multiple activities, we did a secondary cost analysis of nurse-midwives performing EIMC integrated with other routine health services at the primary health care level versus a vertical approach.

## METHODS

In this cost analysis, we sought to estimate the unit costs of EIMC performed by nurse-midwives in comparison with doctor-performed EIMC, and identify the key cost contributors. We collected cost data for nurse-midwives as part of a field trial (n = 500) which assessed the safety, acceptability, and feasibility of EIMC by nurse-midwives using the AccuCirc device.^20^ We then compared these data with cost data for doctor-performed EIMC that had been collected as part of a randomized noninferiority trial that took place before the field trial.^21^ Noninferiority trials test whether a new experimental treatment or strategy is not less efficacious than a treatment already in use.^22^ In this earlier trial, we compared the safety, acceptability, feasibility, and cost profiles of 2 EIMC devices, the AccuCirc and the Mogen clamp.^21^ For the current cost analysis, we used cost data for a doctor-led EIMC procedure using the AccuCirc device. The randomized noninferiority trial and the field study were performed at 2 City of Harare primary care clinics in Mbare and Mabvuku, 2 of Harare’s most populous suburbs. This analysis took the health care payer perspective of Zimbabwe’s Ministry of Health and Child Care (MoHCC) and therefore excludes client costs (transport, absenteeism from work, and caregiver costs).

Results from our companion paper suggest equivalence in safety profiles of EIMC performed by nurse-midwives in comparison with those for doctors.^20^ We therefore adopted cost minimization as the more suitable analysis for this purpose. A cost-minimization analysis defines a situation where, because the consequences of 2 or more treatments or programs are broadly equivalent, the difference between them reduces to a comparison of cost.^23^ We determined all costs in 2014 constant U.S. dollars, and because Zimbabwe officially adopted the U.S. dollar as its principal currency in 2009, our analysis assumes an exchange rate of US$1 = US$1.^24^ Between August 2013 and July 2014, we conducted a detailed cost-data collection exercise using instruments adapted from *Costing Guidelines for HIV Prevention Strategies* from the Joint United Nations Programme on HIV/AIDS (UNAIDS).^25^ We compared these data to those for doctors, collected as part of the earlier randomized noninferiority EIMC pilot trial (January to June 2013) to assess safety, acceptability, feasibility, and cost of AccuCirc compared with the Mogen clamp.^19^

We accessed costs of supplies from invoices and receipts sourced from the respective research partners’ procurement and accounts departments. Nurse-midwives routinely recorded actual resource-use data (drugs and consumable supplies, quantity of devices used, procedure duration, and quantity of waste) for each EIMC procedure on a supplies charge sheet in each infant’s binder. We captured and analyzed direct and indirect cost data using the Decision Makers’ Program Planning Tool (DMPPT), a Microsoft Excel-based model. The United States Agency for International Development (USAID) Health Policy Initiative and UNAIDS developed DMPPT to enable policy makers to analyze costs and impacts of different options for scaling up male circumcision services.^26^

### Direct Costs

#### Supplies and Device Costs

We estimated the total cost of each consumable and nonconsumable drug and supply for 1 EIMC procedure using data on the number of units of each drug or supply needed per procedure and the corresponding unit cost of that drug or supply. The AccuCirc device was obtained for a unit price of US$10 from the international supplier, Clinical Innovations. Since AccuCirc is a single-use device, US$10 was the per-procedure device cost.

#### Personnel Training Costs

A local master trainer (doctor) provided theoretical and practical team training over the course of 5 days for 4 nurse-midwives. Personnel training costs included costs of the time spent by both the master trainer and the nurse-midwives, training material costs, and meal costs. We amortized training costs over a 3.6-year period in line with turnover rates of health care workers in Zimbabwe.^19^^,^^27^ Based on a clinic capacity of 6 EIMC procedures per day and 240 working days in a year (48 weeks and 5 days per week), we estimated that a team (both doctors and nurse-midwives) would perform 1,440 procedures annually. Although the Zimbabwe MoHCC intends to roll out EIMC through nurse-midwives, concerns have been raised about the potential for burnout as they assume additional clinical roles, which may result in time taken off work, additional training costs, and therefore more opportunity costs. To account for potential burnout and avoid underestimating the cost of EIMC delivered by nurse-midwives, we analyzed the cost impact of an annual 2-week leave (and thus 46 instead of 48 full working weeks per year).

#### Personnel Costs

To determine personnel unit costs, we conducted a time and motion study alongside the procedure to capture the time spent by nurse-midwives performing each task for all 500 infants involved in the study, as had been done in the earlier study involving doctors who performed EIMC.^19^ The study team used a stopwatch to time each EIMC procedure and then recorded procedure duration on an EIMC study procedure form. Video recordings were also taken for periodic procedure reviews and time recording. We estimated the total time of an EIMC procedure by averaging time data as captured in procedure forms or through video recordings of each infant's EIMC procedure. We therefore based the total time needed by nurse-midwives to perform an EIMC procedure on direct and continuous observation of the procedure. We defined the beginning of the procedure as the point when the infant’s diaper was removed for cleaning the genital area. The procedure ended when the infant had been bandaged and wrapped up in a new diaper.

To determine personnel unit costs, we conducted a time and motion study alongside the procedure to capture the time spent by nurse-midwives performing each task for all 500 infants involved in the study.

A nurse-midwife salary comprised basic pay plus housing and transport allowances and totaled US$13,596 per year. In the earlier comparative trial, a doctor's salary comprising basic pay and benefits was US$31,800 per year. We derived the total unit cost contribution of personnel to each procedure by summing costs of time for each step of the EIMC procedure for each nurse-midwife. We calculated personnel costs by multiplying the total length of time spent by nurse-midwives performing EIMC by their salary per hour. To estimate the hourly salary rate of a nurse-midwife, we divided the annual salary (monthly salary × 12 months) by the total annual number of hours worked (assuming a 40-hour week × 48 weeks per year). Our primary analysis used research personnel salaries. However, as EIMC rollout is likely to be integrated into routine primary health care services, salary costs are expected to be lower and based on MoHCC salary scales. In sensitivity analysis, we analyzed personnel costs using MoHCC staff salaries, which are lower, in order to assess the cost impacts of different salary scales.

#### Waste Management Costs

To estimate waste management costs, we tracked the number of burn bins (sharps tin containers) used throughout the field trial. We multiplied the total number of burn bins (n = 90) sent to the clinic incinerators by the cost of incineration based on quotes from private service providers (US$10). To determine the contribution to unit cost, we divided the total waste management cost by the number of EIMCs performed in the field trial (n = 500).

### Indirect Costs

#### Capital Costs (Buildings and Durable Equipment)

Because EIMC is likely to be rolled out using existing public health facilities, we excluded costs of buildings. For durable equipment costs, we divided the purchase price of each capital good by an appropriate amortization period to derive an annualized depreciation value. We then summed the annualized depreciation values and divided the total by the estimated annual number of EIMC procedures (n = 1,440) to estimate the unit cost contribution of durable equipment. We charged 100% of the capital cost to EIMC, as capital equipment was exclusively used by EIMC.

#### Support Personnel Costs

The services of support staff (clinic clerk and caretaker) were shared across other clinic services, such as maternity services, antenatal and postnatal services, HIV testing and counseling, pharmacy, outreach, outpatient, and family health services. We therefore apportioned a conservative estimate of 10% of the salaries of both types of personnel as support personnel costs for EIMC. We then divided the summed annual salary costs (including benefits) by the estimated annual number of EIMC procedures (n = 1,440) to derive the unit cost contribution of support personnel.

### Sensitivity Analysis

In one-way sensitivity analysis, we assessed the extent to which the main cost-differentiating components influenced our results by varying each of them, one at a time.^28^^-^^30^ We assessed the unit-cost impacts of using civil service salaries rather than research staff salaries (base case), and whether capacity utilization levels plus procedure duration varied. The capacity utilization analysis aimed to assess the unit cost of EIMCs in cases where staff were unable to reach the daily target of 6 EIMCs (1,440 per year), a possibility in cases of lower demand for EIMC. We also assessed the cost impact of an annual 2-week leave for nurse-midwives to avoid burnout.

### Ethical Considerations

The Medical Research Council of Zimbabwe, the University College London Research Ethics Committee, and the London School of Hygiene and Tropical Medicine Research Ethics Committee approved the EIMC comparative trial and the field study.

## RESULTS

Table 1 summarizes the results for EIMC when performed by nurse-midwives and doctors. The unit cost per EIMC procedure performed by nurse-midwives in a vertical setting was US$38.87 compared with US$49.77 by doctors. Cost differences between nurse-midwives and doctors mainly emanated from personnel and training. Personnel costs were the main contributor to unit costs of EIMC for both nurse-midwives (US$10.57) and doctors (US$19.11). Training had a smaller contribution to unit costs for both nurse-midwives (US$1.51) and doctors (US$3.87). The proportional contribution of personnel declined from 38% for doctors to 27% for nurse-midwives. The unit cost contribution of training also declined from 8% for doctors to 4% for nurse-midwives, and remains constant even when a 2-week annual leave is factored in to account for potential burnout among nurse-midwives. Training costs for nurse-midwives to perform EIMC are therefore lower in comparison with doctors, as the latter cost more per hour.

The unit cost per EIMC procedure by nurse-midwives in a vertical setting was US$38.87 compared with US$49.77 by doctors.

**TABLE 1:** Component Cost of EIMC Performed by Nurse-Midwives and Doctors, 2014

	Nurse-Midwives				Doctors	
	Vertical Program		Integrated Program		Vertical Program	
Cost Category	Cost (US$) per EIMC	% Contribution to Unit Cost	Cost (US$) per EIMC	% Contribution to Unit Cost	Cost (US$) per EIMC	% Contribution to Unit Cost
Direct Costs						
Device	10.00	25.7	10.00	29.7	10.00	20.1
Consumable supplies	13.48	35.0	13.48	40.0	13.48	27.0
Nonconsumable supplies	0.27	0.7	0.27	0.8	0.27	0.5
Waste management	1.80	5.0	1.80	5.0	1.80	4.0
Personnel	10.57	27.0	6.53^a^	19.0	19.11	38.0
Training	1.51	4.0	1.32	4.0	3.87	8.0
**Subtotal (Direct)**	**37.63**	**97.0**	**33.40**	**99.0**	**48.53**	**98.0**
Indirect Costs						
Capital equipment	0.08	0.0	0.00	0.0	0.08	0.0
Support personnel^b^	1.16	3.0	0.32^c^	1.0	1.16	2.0
**Subtotal (Indirect)**	**1.24**	**3.0**	**0.32**	**1.0**	**1.24**	**2.0**
**Grand Total**	**38.87**		**33.72**		**49.77**	

Abbreviation: EIMC, early infant male circumcision.

a Assumes civil servants salary for nurse-midwives.

b Clinic clerk and caretaker.

c Assumes 10% of civil servants salary for caretaker and receptionist.

Costs that were identical for nurse-midwives and doctors included consumable supplies (US$13.48) and nonconsumable supplies (US$0.27), both of which we assumed would remain inflexible even in an integrated setting because they would be dedicated to services offered exclusively in an EIMC department. Other identical costs were the AccuCirc device (US$10.00), waste management (US$1.80), capital equipment (US$0.08), and support personnel (US$1.16).

In secondary analyses, we also compared costs of an EIMC procedure when provided by nurse-midwives in a vertical setting with a procedure provided in a public health facility or clinic (integrated setting). As personnel in a public health facility are employed by the civil service, we also analyzed the impact of using public-sector salaries in the secondary cost analysis. We assumed zero capital equipment costs since setting up EIMC service provision within an existing public health facility would not require new capital equipment. In an integrated public health setting, we found that the unit cost of EIMC was US$33.72 (Table 1). Device, consumable and nonconsumable supplies, waste management, and support personnel costs remained the same as those within the research setting.

In one-way sensitivity analysis (Table 2), we assessed the unit cost impacts of MoHCC salary levels that were below the base case. The unit cost of EIMC procedures performed by nurse-midwives was highly sensitive to personnel costs. Closely related to personnel costs was the time to complete an EIMC procedure. In comparison with a doctor-performed EIMC procedure that took an average of about 16 minutes (rounded up from the exact average of 15.6 minutes) in the comparative trial, results from our companion paper show that nurse-midwives took an average of about 18 minutes (rounded up from the exact average of 17.5 minutes).^20^ In a few cases, however, procedures were either shorter (a minimum of 12 minutes) or longer. In one case, it took much longer to bring the bleeding under control (total duration of the procedure was 48 minutes). In sensitivity analysis (Table 2), we therefore assessed the impact on unit cost of using these outlier durations. For the 12-minute procedure, the cost was US$37.69 (compared with US$38.87 for the 18-minute average time), and for the 48-minute procedure, the cost was US$46.19. We also assessed the impact on unit cost of time observations using the 25%–75% interquartile range (middle 50%) as it was felt that 48 minutes was such a far outlier. For the 13-minute procedure (25th percentile), the cost was US$37.92 (compared with US$38.87 for the 18-minute average time), and for the 30-minute procedure (75th percentile), the cost was US$41.94.

**TABLE 2:** One-Way Sensitivity Analysis of Costs of EIMC Performed by Nurse-Midwives and Doctors, 2014

	EIMC Unit Cost (US$)	
Cost Category	Nurse-Midwives	Doctors
Personnel salaries		
Base case (study salaries)	38.87	49.77
Public sector^a^ (integrated)	34.00	N/C
Capacity utilization (EIMC/day)		
2	44.36	N/C
4	40.23	N/C
6 (base case)	38.87	49.77
8	38.18	N/C
Staff time for EIMC procedure (using outlier times observed), minutes		
12	37.69	N/C
18 for nurse-midwives; 16 for doctors (base case)	38.87	49.77
48	46.19	N/C
Staff time for EIMC procedure (using 25%–75% interquartile range), minutes		
13 (25th percentile)	37.92	N/C
18 for nurse-midwives; 16 for doctors (base case)	38.87	49.77
30 (75th percentile)	41.94	N/C
Effect of nursing staff leave (due to burnout), weeks		
46	38.93	N/C
48 (base case)	38.87	49.77

Abbreviations: EIMC, early infant male circumcision; N/C, no change.

a Public-sector salaries inclusive of benefits are US$26,400 for a doctor, US$8,400 for a nurse-midwife, US$3,000 for a receptionist, and US$1,800 for a caretaker.

We found the largest cost difference between vertical and integrated EIMC in personnel costs (US$10.57 vs. US$6.53, respectively) (Table 1). Training costs were also lower in the integrated model of EIMC delivery (US$1.51 vs. US$1.32), as were support personnel costs (US$1.16 vs. US$0.32), because we assumed that they were shared across other clinic services. We also assessed the effect of capacity utilization and staff time but found that the EIMC unit cost was not sensitive to either one.

The largest cost difference between vertical and integrated EIMC by nurse-midwives was found in personnel costs (US$10.57 vs. US$6.53).

## DISCUSSION

Substituting nurse-midwives for doctors presents considerable opportunities for minimizing costs in a future EIMC rollout, without impacting safety. Our results show that in a vertical EIMC program it would cost substantially less to perform an EIMC procedure in Zimbabwe using nurse-midwives compared with doctors. Further opportunities for cost savings exist if EIMC were rolled out using an integrated model because civil service medical personnel costs are lower. In our analysis, personnel costs for EIMC performed by nurse-midwives fall between US$10.57 in a vertical program and US$6.53 in an integrated program (assuming use of civil service salaries). A combination of these factors, therefore, makes it more feasible for MoHCC policy makers to meet the goal of rolling out the EIMC procedure more widely and at a lower cost.

This cost analysis of EIMC performed by nurse-midwives and doctors is, to the best of our knowledge, the first analysis based on actual costs of providing EIMC using nurse-midwives in sub-Saharan Africa. For other contexts, actual EIMC costs may differ from those described in this analysis. In a hypothetical modeling study conducted in Rwanda, EIMC was estimated to cost US$15 (direct costs only) using the Mogen clamp.^4^ Using similar assumptions (including integration of EIMC in routine primary health services), our analysis of actual costs using AccuCirc yielded a unit cost of US$33.72, which is much higher than that for Rwanda. In our study, however, we used a greater number of inputs, including staff, essential consumables, and consumables used during 3 follow-up visits, whereas the Rwanda study limited the costing to the actual circumcision procedure.

This study has at least 3 limitations that influence whether the costs reported here would be the same as those in a scaled-up program. First, EIMC procedures were performed in an urban setting, and thus the results may be different for rural settings. Second, we based this cost analysis on a pilot implementation among a sample of 500 infants. To facilitate our analysis, we assumed that each team of nurse-midwives would perform 1,440 EIMC procedures each year. While this number was based on conservative assumptions, lower demand for EIMC may lead to lower capacity utilization resulting in higher unit costs of EIMC. Finally, our analyses did not include costs of demand creation due to the short duration (6 months) of the field study and the small sample size. Including realistic estimates of demand creation will be essential for fully costing a scaled-up EIMC program.

The potential impact of VMMC in reducing the number of new HIV infections has led some countries in sub-Saharan Africa to consider rolling out EIMC in order to achieve higher prevalence of male circumcision in the medium to long term.^8^^,^^21^ However, the shortage of medical personnel, particularly doctors, threatens the feasibility of these strategies and has led policy makers to consider substituting nurse-midwives for doctors to scale up EIMC. An understanding of the unit cost of EIMC performed by nurse-midwives can aid policy makers in decision making regarding the optimal delivery models to use. Despite its limitations, therefore, this study along with data from a companion study,^21^ provides valuable data for planning EIMC scale-up and illustrates the advantages of using a nurse-midwife model. It also highlights that an integrated EIMC program is cost-minimizing compared with a vertical program, and helps determine the overall resources needed to implement a scaled-up EIMC program. Nurse-midwives could facilitate widespread low-cost scale-up of EIMC in sub-Saharan Africa. However, as nurse-midwives are currently overburdened, this strategy can only succeed if additional nurse-midwives are trained.

Our study highlights that an integrated EIMC program is cost-minimizing compared with a vertical program and helps to determine the overall resources needed to implement a scaled-up EIMC program.

## Notes

**Peer Reviewed**
